# Health Outcome Comparison Based on Dietary Inflammatory Levels among Sample of Korean Elderly

**DOI:** 10.3390/healthcare12101003

**Published:** 2024-05-13

**Authors:** Seul-Ki Koo, Hee-Sook Lim

**Affiliations:** Department of Gerontology, AgeTech-Service Convergence Major, Graduate School of East-West Medical Science, Kyung Hee University, Yongin 17104, Republic of Korea

**Keywords:** inflammation, senior friendly diet, nutritional status, health, hematological markers

## Abstract

The aim of this study was to investigate the effect of a senior-friendly diet based on the dietary inflammatory index (DII) on the nutritional status and health indicators of older people. A total of 256 participants were classified into tertiles based on their DII values and split into intervention *(n* = 201) and control (*n* = 55) groups. The intervention group was provided with a senior-friendly diet, and the control group was allowed to eat their usual diet. Before and after the trial, anthropometric measurements, blood analyses, and questionnaires were completed for both groups. The mean age of the participants was 82.49 years, and 66.4% were female. On average, participants had 2.5 medical conditions, with a notable prevalence of cardiovascular disease. Following the intervention, the energy, carbohydrate, protein, and fat intakes significantly increased in the intervention group compared to pre-intervention levels. Tertile 3 exhibited substantial improvements in total nutrition score, NQ-E balance, and DII total score, as well as in triglycerides and blood glucose, attributed to this dietary intervention compared to other groups. In comparison with Tertile 1, nutrient intake and nutritional status in Tertile 3 were closely associated with significant influencing factors of the dietary intervention. For the group with the worst DII (Tertile 3), this dietary intervention greatly improved nutritional status, nutrient intake, and clinical indicators; thus, this senior-friendly diet appears to be beneficial for elderly people with nutritional vulnerabilities.

## 1. Introduction

Aging populations are rapidly increasing worldwide [1]. For instance, the average life expectancy in South Korea has increased due to advances in medical technology and improvements in living standards [2]. By 2023, the population ≥65 years of age in South Korea is estimated to reach 9.5 million, accounting for approximately 18.4% of the total population. This is projected to become approximately 21% by 2025. Therefore, South Korea is progressing toward a superaged society at a faster rate than other countries [3]. Aging is associated with a low-grade chronic inflammatory state, known as inflammaging [4]. Inflammation is a biological response to tissue damage or infection [5]. Although appropriate inflammatory responses maintain homeostasis, their dysregulation can lead to social impairment and the development of chronic diseases. Further, although inflammation [6] is influenced by environmental factors and genetic background [7], the relationship between dietary factors, such as nutrients, foods, and dietary patterns, and the inflammatory response remains unclear.

The dietary inflammatory index (DII) reflects the inflammatory potential of an individual’s diet, allowing the assessment of the relationship between dietary factors and inflammation in the context of disease progression. It was developed based on epidemiological studies, animal experiments, and cell studies that indicated associations between the levels of inflammatory markers such as Interleukin-1 beta (IL-1β), Interleukin-4 (IL-4), Interleukin-6 (IL-6), Interleukin-10 (IL-10), Tumor Necrosis Factor-alpha (TNF-α), and C-reactive protein (CRP) with 36 nutrients and 9 food groups [8]. The DII score is calculated as described previously [9], and the mean and SD values are used to obtain Z-scores. A higher DII score indicates a more proinflammatory diet, whereas a lower score indicates a more anti-inflammatory diet [10]. DII score is reportedly associated with a range of health conditions, including metabolic disorders, cardiovascular disease [11], asthma, pancreatic cancer, colorectal cancer, and neurocognitive disorders [12]. In fact, longitudinal cohort studies have shown that higher DII scores are associated with an increased risk of obesity, as assessed by body mass index (BMI), waist circumference, and waist-to-hip ratio [13]. Moreover, higher DII scores have been associated with increased rates of cardiovascular disease, cancer, and gastrointestinal mortality [9]. Although previous studies have involved diverse age groups and large cohorts, so far few have focused on DII in adults ≥65 years of age in South Korea. Furthermore, no study has evaluated the elderly population from a life-course perspective.

Proper nutritional management is essential for older adults to maintain their health status [14]. However, age-related chewing difficulties, reduced digestive function, and other factors related to food intake can lead to nutritional imbalance [15] and increase the prevalence of chronic diseases [16]. Therefore, the food properties related to chewing, swallowing, and digestion are particularly important; further, there is an increasing need for senior-friendly foods that excel in nutritional value, convenience, and palatability [16]. In South Korea, the Ministry of Agriculture, Food, and Rural Affairs established standards for senior-friendly industries in 2017; the Korean Senior-Friendly Food Specification (KS H 4897) promotes the development and provision of foods that address common nutritional imbalances in older adults. Meal consumption is an important part of the daily life of older adults.

Therefore, we investigated the correlation between the DII and positive changes in clinical indicators in individuals aged ≥65 years who consumed senior-friendly nutritious foods.

## 2. Materials and Methods

### 2.1. Study Participants

After participants were fully informed about the study, informed consent was obtained from them or their guardians. The study procedures were approved by the Institutional Review Board of Kyung Hee University (KHGIRB-22-206). The study, funded by the National Food Cluster Promotion Institute, was conducted from April to December, totaling 8 months, with the actual dietary intervention taking place from June to October, totaling 5 months. In total, 295 participants took part in the study, comprising 204 community-dwelling older adults utilizing non-graded comprehensive care services and home-visiting care services, and 91 older adults residing in day and night care institutions. The number of participants was determined based on a previously established research model in Korea [17]. There are two reasons for the differences between the intervention and control groups. Firstly, as no previous research results were found using the Pass 2022 program, we adopted a similar model of study to estimate the rate of change in nutritional status, resulting in an approximate 4% rate of change being applied. Secondly, the funding institution proposed the research within its resource scope. Participants were randomly assigned to the intervention and control groups to investigate the effects of a high-quality, age-appropriate diet. DII scores were used to categorize participants into groups prior to pre- and post-intervention comparisons. At the conclusion of the study, 24 community-dwelling older adults were excluded due to withdrawal. This included withdrawals (15 participants), refusals (8 participants), discontinuation for more than 45 days (8 participants), and death (1 participant). Among older adults residing in institutions, 15 were excluded due to hospitalization (10 participants), relocation (1 participant), and discharge (4 participants). In total, the study included 256 participants, with 201 in the intervention group and 55 in the control group.

### 2.2. General Characteristics

In this study, general characteristics such as age, sex, household type, educational level, monthly income, alcohol consumption, smoking status, BMI, Korean Activities of Daily Living (K-ADL) score, and average number of diseases were evaluated. Participants were divided into 65–74, 75–84, and ≥85 age groups. Household type was categorized as living alone or with others, and educational level was categorized as no education, primary school, middle school, high school, or university or higher. BMI [18] is an index used to assess an individual’s body fat percentage by using their weight and height. Based on guidelines established by health organizations such as the World Health Organization (WHO), this study classified BMI into four categories according to standards in the Asia–Pacific region [19]. The adequacy of activities of daily living (ADL) was assessed using the K-ADL tool.

### 2.3. Dietary Intervention

We divided the participants into intervention and control groups and provided meals accordingly. The control group maintained their existing meals provided by the institutions, while the intervention group received replacements with senior-friendly diets.

### 2.4. DII Score and Nutrient Intake

Dietary intake was assessed using a 24 h dietary recall method by registered dietitians who conducted home or facility visits. Participants were asked to recall and provide detailed information on the types, quantities, ingredients, and cooking methods of all foods consumed during the previous day. Data were collected for three consecutive days for both the pre- and postintervention periods, and analyzed using Can-Pro 5.0 software (Computer-Aided Nutritional Analysis Program for Professionals, The Korean Nutrition Society, Seoul, Republic of Korea) to determine nutrient intake [20].

The DII comprises 45 variables, including pro- and anti-inflammatory nutrients, flavonoids, spices, and foods [9]. We measured carbohydrates, total fat, protein, fiber, vitamin A, β-carotene, vitamin D, vitamin E, vitamin C, thiamine, riboflavin, niacin, vitamin B_6_, folic acid, vitamin B_12_, Mg, Fe, Zn, Se, cholesterol, saturated fat, monounsaturated fatty acids (MUFAs), polyunsaturated fatty acids (PUFAs), n-3 fatty acids, alcohol, caffeine, garlic, ginger, onion, turmeric, pepper, and green/black tea. Eleven substances—eugenol, rosemary, saffron, thyme/oregano, flavan-3-ol, flavones, flavanols, flavanones, anthocyanidin, and isoflavones—not commonly consumed by South Koreans were not included in this study. The DII was developed on the basis of epidemiological studies, animal experiments, and cellular studies that indicated relationships among inflammatory markers, nutrients, and foods. It includes 36 final nutrients and 9 food items as indicators. To determine the DII score, the intake of each indicator was calculated using 33 intermediate parameters. The participants were divided into Tertile 1 (−0.817–1.308), Tertile 2 (1.310–2.572), and Tertile 3 (2.578–6.553). Based on the participants’ analyzed values, Tertile 1 represents the group with low dietary inflammatory index (DII) values, Tertile 2 represents the intermediate group, and Tertile 3 represents the group with high DII values.

### 2.5. DETERMINE Checklist and Nutrition Quotient for Elderly Score

The DETERMINE checklist was used to assess the nutritional status of community-dwelling older adults. It consists of the following indicators: illness, poor diet, tooth loss/mouth pain, economic hardship, reduced social contact, multiple medications, involuntary weight loss/gain, need for help with self-care [14], and age >80 years [21]. To determine their nutritional adequacy, individuals were categorized based on their average score as low risk (0–2 points), moderate risk (3–5 points), or high risk (≥6 points). The nutrition quotient for the elderly (NQ-E) is a validated questionnaire in South Korea to assess the diet quality of older adults. It consists of four domains—food behavior, balance, diversity, and moderation—with nineteen items. Mean scores were calculated for balance, variety, moderation [22], dietary behavior, and total dietary score [23].

### 2.6. Clinical Examination

Blood samples were obtained to assess the appropriateness of the clinical examinations. Blood pressure as well as triglycerides, total cholesterol, LDL cholesterol, HDL cholesterol, blood glucose, hemoglobin, and CRP were measured in the blood. The Afinion2 analyzer (Abbott, Norway) was used for HbA1c, lipids, and CRP panels, the Hemochroma Plus (Boditech Med, Seoul, Republic of Korea) for hemoglobin (mg/dL) and hematocrit (%) measurements, and the GlucoDr. GOLD (Greenbiomedical, Seoul, Republic of Korea) for blood glucose (mg/dL). The blood test results were examined to determine the appropriateness of the clinical diagnostic criteria.

### 2.7. Statistical Analysis

Data analyses were performed using the Statistical Package for SPSS (version 25.0). Between-group differences in participant characteristics and secondary outcome measures were assessed using the Mann–Whitney U test for continuous variables and the chi-square test for categorical variables. Multiple regression analysis was performed to analyze the factors affecting the DII index. We performed a mean centering of the independent variables, control variables, and interaction variables to confirm the lack of a multicollinearity problem through the variance inflation factor. *p* < 0.05 indicated statistical significance.

## 3. Results

### 3.1. Characteristics of the Participants

The average age of the participants was 82.49 years, with 170 females (66.4%). A total of 189 participants (73.8%) were living alone, and among them, 187 (73.0%) had no education or elementary school education as their educational level. The average BMI of the participants was 23.3 kg/m^2^, indicating overweight status. In addition, 216 participants had a monthly income of less than 84.4%. With regard to alcohol consumption and smoking, 224 (87.5%) and 242 (94.5%) participants reported that they did not drink or smoke, respectively, indicating that the majority did not drink or smoke. The participants had a K-ADL score of 8.3, indicating no difficulty in performing activities of daily living independently. The average disease occurrence rate among the participants was 2.5, with a high prevalence of cardiovascular diseases. There were no significant differences in baseline characteristics between the groups (Table 1).

### 3.2. Nutrient Intake According to Dietary Intervention in the DII Group

The actual nutrient intake of the participants was compared with that recommended by the Korean Dietary Reference Intakes for Koreans aged 75 years and older [20]. Overall, despite deficiencies compared to the recommended levels, there was an improvement in nutrient intake. In terms of energy, women met the requirements, but men did not. Both genders met the requirements for carbohydrates, protein, fiber, vitamin A, β-carotene, vitamin E, thiamin, riboflavin, vitamin B_6_, iron, zinc, saturated fatty acids, and cholesterol, but did not meet the requirements for vitamin D, vitamin C, niacin, folic acid, vitamin B_12_, magnesium, or selenium (Table 2).

### 3.3. Nutritional Status and Diet Quality According to Dietary Intervention in the DII Group

The average preintervention DETERMINE score of the participants decreased from 6.3 to 5.0, indicating a reduction in nutritional risk from high to moderate. All intervention groups showed a significantly lower risk compared with the control group. The NQ-E scores for nutrition, balance, variety, moderation, and dietary behavior improved after the intervention. Among the intervention groups, significant improvements were observed in nutrition, variety, and dietary behavior in Tertile 1; in nutrition, balance, variety, and dietary behavior in Tertile 2; and in nutrition, balance, and dietary behavior in Tertile 3. In contrast, the control group did not show significant improvements in any categories (Table 3).

### 3.4. Dietary Inflammatory Index and Clinical Examination According to Dietary Intervention in the DII Group

The post-DII score improved significantly in both the intervention and control groups. The difference in improvement was greatest in Tertile 3, with −5.1 points in the intervention group and −3.8 points in the control group. In addition, the total cholesterol level improved significantly in the intervention group, particularly in Tertile 3, which had a poor DII score pre-treatment. Blood glucose levels significantly improved from 146.9 to 135.9 mg/dL in Tertile 3. However, there was no significant difference in CRP levels between the groups (Table 4).

**Table 2:** Comparison of nutrient intake according to dietary intervention by DII group.

Variables	Total *n* = 256	Tertile 1 *n* = 85				Tertile 2 *n* = 85				Tertile 3 *n* = 85			
		Intervention Group *n* = 67		Control Group *n* = 18		Intervention Group *n* = 67		Control Group *n* = 18		Intervention Group *n* = 67		Control Group *n* = 19	
	Post (Pre)	Post (Pre)	*p*-Value	Post (Pre)	*p*-Value	Post (Pre)	*p*-Value	Post (Pre)	*p*-Value	Post (Pre)	*p*-Value	Post (Pre)	*p*-Value
Energy (Kcal)	1570.1 ± 209.9 (1437.2 ± 205.0)	1612.9 ± 181.7 (1482.6 ± 153.8)	0.000	1529.1 ± 118.4 (1562.3 ± 125.2)	0.120	1611.8 ± 213.1 (1473.8 ± 161.4)	0.000	1514.5 ± 93.6 (1524.7 ± 174.5)	0.916	1549.9 ± 234.5 (1290.5 ± 247.0)	0.000	1435.5 ± 218.5 (1463.5 ± 169.7)	0.505
Carbohydrate (g)	228.1 ± 32.8 (231.4 ± 35.1)	235.8 ± 27.4 (222.7 ± 38.3)	0.016	224.2 ± 24.2 (236.7 ± 34.4)	0.268	228.9 ± 33.9 (243.1 ± 28.1)	0.015	221.0 ± 34.1 (240.4 ± 21.9)	0.098	227.6 ± 36.0 (223.6 ± 38.8)	0.560	211.0 ± 35.6 (235.1 ± 30.9)	0.012
Protein (g)	61.0 ± 8.8 (50.9 ± 12.4)	64.0 ± 7.7 (58.7 ± 10.1)	0.000	53.5 ± 7.1 (57.0 ± 8.3)	0.005	63.7 ± 7.5 (50.9 ± 9.5)	0.000	54.2 ± 6.3 (52.2 ± 6.0)	0.251	61.7 ± 9.2 (42.0 ± 14.4)	0.000	41.0 ± 7.8 (35.1 ± 11.2)	0.008
Fat (g)	43.6 ± 8.8 (32.6 ± 15.3)	44.0 ± 8.9 (38.7 ± 14.0)	0.004	44.2 ± 5.3 (41.8 ± 4.6)	0.439	45.6 ± 9.8 (31.3 ± 14.5)	0.000	44.2 ± 6.8 (37.7 ± 13.6)	0.014	41.5 ± 8.7 (23.4 ± 14.4)	0.000	52.0 ± 6.3 (47.4 ± 5.8)	0.024
Fiber (g)	20.7 ± 10.0 (22.4 ± 6.9)	21.3 ± 10.8 (28.5 ± 5.6)	0.000	20.4 ± 1.8 (25.9 ± 3.4)	0.000	19.3 ± 3.2 (22.5 ± 3.9)	0.000	20.5 ± 2.5 (21.5 ± 2.0)	0.070	22.1 ± 15.8 (16.1 ± 6.8)	0.007	19.0 ± 3.4 (20.5 ± 4.7)	0.315
Vitamin A (ug)	368.1 ± 119.1 (473.0 ± 298.8)	380.2 ± 115.0 (733.9 ± 302.9)	0.000	373.5 ± 125.0 (590.9 ± 228.1)	0.000	344.5 ± 111.0 (481.4 ± 230.1)	0.000	323.6 ± 96.7 (453.0 ± 193.4)	0.011	396.2 ± 121.6 (222.6 ± 166.4)	0.000	346.7 ± 145.8 (313.3 ± 142.9)	0.475
β-Carotene (ug)	3669.5 ± 1103.2 (5282.6 ± 3653.2)	3777.4 ± 1109.9 (8506.2 ± 3733.9)	0.000	3799.1 ± 1135.4 (6757.5 ± 2779.7)	0.000	3400.8 ± 954.9 (5370.5 ± 2748.5)	0.000	3391.4 ± 903.9 (5042.6 ± 2477.5)	0.005	3899.4 ± 1101.0 (2217.9 ± 1919.5)	0.000	3566.6 ± 1527.7 (3242.8 ± 1830.9)	0.552
Vitamin D (ug)	3.2 ± 2.3 (1.4 ± 2.2)	3.4 ± 2.6 (1.3 ± 3.2)	0.000	2.4 ± 1.9 (0.6 ± 0.5)	0.004	2.9 ± 2.2 (1.5 ± 2.2)	0.000	2.5 ± 2.2 (0.9 ± 1.0)	0.001	3.7 ± 2.0 (1.5 ± 1.5)	0.000	2.8 ± 2.4 (1.7 ± 1.4)	0.026
Vitamin E (mg)	16.8 ± 5.3 (12.1 ± 4.7)	15.7 ± 4.8 (15.3 ± 3.8)	0.556	20.9 ± 4.0 (14.9 ± 5.1)	0.001	17.3 ± 5.8 (11.4 ± 2.8)	0.000	20.7 ± 3.6 (11.5 ± 3.0)	0.000	14.7 ± 4.8 (9.2 ± 5.5)	0.000	18.8 ± 4.3 (11.1 ± 2.5)	0.000
Vitamin C (mg)	65.7 ± 16.8 (64.2 ± 35.3)	67.7 ± 7.5 (70.5 ± 39.1)	0.606	65.5 ± 12.1 (92.0 ± 30.7)	0.005	64.2 ± 15.6 (73.2 ± 27.2)	0.022	66.6 ± 12.6 (80.0 ± 27.7)	0.252	65.2 ± 19.3 (38.6 ± 28.3)	0.000	65.0 ± 17.8 (59.6 ± 29.5)	0.516
Thiamin (mg)	1.3 ± 0.3 (1.5 ± 0.5)	1.3 ± 0.3 (1.7 ± 0.4)	0.000	1.5 ± 0.1 (1.9 ± 0.4)	0.000	1.3 ± 0.3 (1.5 ± 0.5)	0.000	1.5 ± 0.2 (1.7 ± 0.4)	0.015	1.2 ± 0.2 (1.1 ± 0.5)	0.179	1.4 ± 0.2 (1.6 ± 0.4)	0.112
Riboflavin (mg)	1.3 ± 4.2 (1.0 ± 0.4)	2.0 ± 8.3 (1.0 ± 0.3)	0.337	1.1 ± 0.2 (1.2 ± 0.3)	0.185	1.0 ± 0.2 (1.1 ± 0.3)	0.201	1.0 ± 0.2 (1.2 ± 0.3)	0.147	1.1 ± 0.2 (0.8 ± 0.4)	0.000	1.1 ± 0.3 (1.1 ± 0.3)	0.944
Niacin (mg)	9.4 ± 1.6 (10.5 ± 3.5)	9.4 ± 1.6 (12.1 ± 2.7)	0.000	9.9 ± 1.2 (12.1 ± 4.6)	0.040	9.2 ± 1.7 (10.5 ± 2.4)	0.000	9.6 ± 1.4 (11.0 ± 3.6)	0.092	9.2 ± 1.8 (8.7 ± 4.2)	0.328	9.4 ± 1.8 (9.4 ± 1.9)	0.924
Vitamin B_6_ (mg)	1.6 ± 0.8 (1.2 ± 0.6)	1.4 ± 0.4 (1.5 ± 1.0)	0.780	2.2 ± 1.5 (1.4 ± 0.2)	0.043	1.3 ± 0.3 (1.3 ± 0.2)	0.140	2.4 ± 1.5 (1.3 ± 0.2)	0.007	1.4 ± 0.5 (0.9 ± 0.4)	0.000	2.0 ± 1.3 (1.2 ± 0.2)	0.013
Folic acid (mg)	432.6 ± 92.9 (391.9 ± 119.7)	437.7 ± 92.4 (446.9 ± 111.5)	0.550	429.2 ± 80.0 (478.3 ± 97.7)	0.020	419.9 ± 90.8 (403.4 ± 99.5)	0.337	414.6 ± 66.5 (391.0 ± 80.4)	0.554	452.6 ± 107.4 (309.8 ± 126.7)	0.000	409.1 ± 71.8 (365.3 ± 44.4)	0.072
Vitamin B_12_ (mg)	6.4 ± 2.9 (3.9 ± 4.1)	6.3 ± 2.5 (4.6 ± 4.2)	0.006	5.2 ± 2.9 (4.3 ± 3.7)	0.381	6.4 ± 3.2 (4.1 ± 3.3)	0.000	4.5 ± 2.2 (4.0 ± 2.4)	0.075	7.6 ± 2.7 (3.3 ± 5.5)	0.000	5.8 ± 3.2 (2.1 ± 1.9)	0.000
Magnesium (mg)	243.0 ± 26.3 (106.4 ± 51.4)	209.7 ± 51.4 (115.5 ± 42.8)	0.348	78.9 ± 13.2 (115.6 ± 36.3)	0.000	75.9 ± 18.6 (117.9 ± 57.1)	0.000	82.5 ± 5.7 (105.7 ± 36.8)	0.026	76.7 ± 16.2 (86.6 ± 58.3)	0.204	80.4 ± 19.8 (95.7 ± 35.5)	0.075
Iron (mg)	14.0 ± 2.7 (13.6 ± 4.2)	14.3 ± 2.7 (16.1 ± 3.4)	0.002	14.5 ± 1.3 (15.4 ± 1.7)	0.100	13.8 ± 2.7 (14.0 ± 3.0)	0.609	14.3 ± 2.1 (13.8 ± 1.6)	0.730	13.9 ± 3.3 (10.8 ± 5.4)	0.000	13.8 ± 2.1 (11.5 ± 1.7)	0.001
Zinc (mg)	7.3 ± 1.4 (8.8 ± 2.1)	7.3 ± 1.1 (10.3 ± 1.8)	0.000	8.1 ± 2.0 (9.9 ± 1.1)	0.000	7.0 ± 1.1 (9.2 ± 1.2)	0.000	8.4 ± 0.9 (9.5 ± 0.5)	0.001	7.1 ± 1.3 (6.7 ± 1.9)	0.152	7.7 ± 1.8 (8.3 ± 1.2)	0.323
Saturated fat (g)	8.6 ± 2.3 (8.1 ± 5.2)	8.5 ± 1.8 (11.1 ± 5.9)	0.002	8.5 ± 1.7 (10.5 ± 5.0)	0.183	8.7 ± 1.7 (7.4 ± 1.7)	0.030	8.4 ± 1.9 (8.8 ± 4.6)	0.741	8.9 ± 3.4 (4.7 ± 3.1)	0.000	7.9 ± 1.1 (8.9 ± 4.3)	0.359
Cholesterol (g)	486.3 ± 191.2 (243.1 ± 95.6)	519.7 ± 176.7 (260.0 ± 88.4)	0.000	430.9 ± 169.2 (215.5 ± 84.6)	0.000	496.0 ± 201.6 (248.0 ± 100.8)	0.000	378.9 ± 192.1 (189.4 ± 96.0)	0.000	498.1 ± 193.1 (249.0 ± 96.5)	0.000	446.9 ± 185.3 (223.4 ± 92.7)	0.000

DII score range: Tertile 1 (−0.817~1.308), Tertile 2 (1.310~2.572), Tertile 3 (2.578~6.553). Parentheses are baseline results. Data are reported as mean ± standard deviation for continuous variables and as percentages for categorical variable. *p*-value was calculated by Student’s *t*-test or Mann–Whitney U test for continuous variables as appropriate.

**Table 3:** Comparison of nutritional status and quality of diet according to dietary intervention by DII group.

Variables		Total *n* = 256		Tertile 1 *n* = 85						Tertile 2 *n* = 85						Tertile 3 *n* = 85					
				Intervention Group *n* = 67			Control Group *n* = 18			Intervention Group *n* = 67			Control Group *n* = 18			Intervention Group *n* = 67			Control Group *n* = 19		
		Pre	Post	Pre	Post	*p*-Value	Pre	Post	*p*-Value	Pre	Post	*p*-Value	Pre	Post	*p*-Value	Pre	Post	*p*-Value	Pre	Post	*p*-Value
Nutritional status	Score	6.3 ± 3.9	5.0 ± 3.9	7.1 ± 3.7	5.5 ± 4.1	0.000	4.6 ± 3.5	3.3 ± 0.0	0.058	5.8 ± 3.8	4.5 ± 3.6	0.000	5.2 ± 4.4	3.8 ± 4.3	0.062	7.2 ± 3.8	5.9 ± 4.0	0.000	5.2 ± 4.0	4.3 ± 3.9	0.128
	Well-nourished	20.3	36.7	14.9	32.8		33.3	50.0		25.4	41.8		27.8	55.6		11.9	23.9		31.6	47.4	
	At risk of malnutrition	22.7	21.9	17.9	19.4		27.8	22.2		23.9	16.4		38.9	27.8		19.4	29.9		26.3	15.8	
	Malnourished	57.0	41.4	67.2	47.8		38.9	27.8		50.7	41.8		33.3	16.7		68.7	46.3		42.1	36.8	
NQ-E	Total nutrition score	47.7 ± 9.0	51.0 ± 9.5	46.3 ± 8.4	50.0 ± 9.4	0.000	52.7 ± 8.2	55.0 ± 8.8	0.090	48.6 ± 9.9	53.1 ± 10.7	0.000	49.4 ± 8.2	51.9 ± 7.3	0.115	45.2 ± 7.5	47.7 ± 7.5	0.028	52.2 ± 10.6	54.4 ± 10.3	0.211
	Balance	22.6 ± 17.4	25.8 ± 18.6	23.9 ± 18.5	27.9 ± 17.1	0.819	24.4 ± 18.8	34.3 ± 20.3	0.076	21.1 ± 16.5	28.0 ± 18.1	0.001	22.1 ± 12.9	23.4 ± 16.3	0.715	20.4 ± 16.7	21.8 ± 17.0	0.009	26.4 ± 22.0	31.0 ± 23.0	0.115
	Diversity	23.1 ± 12.6	24.7 ± 12.6	22.1 ± 12.2	24.5 ± 13.4	0.022	31.0 ± 12.1	30.0 ± 11.2	0.529	23.7 ± 14.2	26.2 ± 13.8	0.003	28.9 ± 12.3	28.6 ± 12.0	0.885	19.4 ± 9.5	20.7 ± 9.8	0.694	24.7 ± 13.8	25.6 ± 13.5	0.762
	Moderation	87.9 ± 14.4	89.7 ± 13.1	86.4 ± 15.5	89.5 ± 10.7	0.057	89.0 ± 13.1	90.0 ± 10.4	0.809	80.1 ± 14.5	91.4 ± 12.9	0.107	83.2 ± 14.1	88.5 ± 13.9	0.180	88.2 ± 14.4	88.1 ± 14.2	0.165	91.5 ± 13.4	92.3 ± 11.4	0.719
	Dietary behavior	40.6 ± 16.5	46.8 ± 17.4	36.9 ± 14.1	46.9 ± 15.1	0.000	45.0 ± 16.5	50.6 ± 16.3	0.163	42.9 ± 19.6	49.5 ± 19.8	0.000	47.6 ± 13.4	49.8 ± 16.9	0.526	36.1 ± 14.1	42.7 ± 15.6	0.027	48.6 ± 17.4	51.4 ± 17.5	0.258

DII score range: Tertile 1 (−0.817~1.308), Tertile 2 (1.310~2.572), Tertile 3 (2.578~6.553). Nutrition Quotient for Elderly (NQ-E).

**Table 4:** Comparison of dietary inflammatory index score and clinical examination according to dietary intervention by DII group.

Variables	Total N = 256		Tertile 1 *n* = 85						Tertile 2 *n* = 85						Tertile 3 *n* = 85					
			Intervention Group *n* = 67			Control Group *n* = 18			Intervention Group *n* = 67			Control Group *n* = 18			Intervention Group *n* = 67			Control Group *n* = 19		
	Pre	Post	Pre	Post	*p*-Value	Pre	Post	*p*-Value	Pre	Post	*p*-Value	Pre	Post	*p*-Value	Pre	Post	*p*-Value	Pre	Post	*p*-Value
DII score	2.1 ± 1.7	−0.7 ± 1.2	0.4 ± 0.6	−0.7 ± 1.1	0.000	0.7 ± 0.6	−0.5 ± 1.5	0.001	2.0 ± 0.4	−0.5 ± 1.1	0.000	1.9 ± 0.3	−0.7 ± 1.7	0.000	4.2 ± 1.3	−1.0 ± 0.9	0.000	3.0 ± 1.0	−0.5 ± 1.6	0.000
	(−2.8 ± 2.2)		(−1.2 ± 1.1)			(−1.2 ± 1.3)			(−2.5 ± 1.4)			(−20.6 ± 10.7)			(−5.1 ± 1.9)			(−3.8 ± 2.1)		
Triglycerides (mg/dL)	166.6 ± 77.9	151.6 ± 69.6	166.7 ± 77.5	160.1 ± 74.4	0.362	207.8 ± 91.6	200.2 ± 54.1	0.688	161.6 ± 62.6	143.3 ± 68.6	0.018	163.4 ± 101.0	146.5 ± 80.2	0.128	162.6 ± 84.4	143.2 ± 67.6	0.013	164.2 ± 66.0	143.1 ± 49.2	0.084
≥150	50.4	55.9	49.3	56.7		66.7	83.3		50.7	40.3		27.8	38.9		46.3	40.3		63.2	42.1	
Total cholesterol (mg/dL)	159.8 ± 39.2	148.8 ± 36.2	159.3 ± 44.8	148.3 ± 37.8	0.004	183.8 ± 49.9	166.8 ± 44.9	0.058	158.4 ± 32.7	149.3 ± 32.0	0.000	162.6 ± 30.8	155.4 ± 37.5	0.059	152.9 ± 38.6	139.4 ± 36.5	0.000	166.0 ± 31.4	158.7 ± 25.5	0.143
≥200	14.1	9.8	17.9	11.9		27.8	11.1		9.0	7.5		11.1	16.7		13.4	7.5		10.5	10.5	
LDL cholesterol (mg/dL)	76.2 ± 33.0	73.9 ± 31.3	81.5 ± 34.6	78.7 ± 32.4	0.347	89.1 ± 39.5	83.1 ± 35.3	0.547	71.4 ± 29.5	70.5 ± 27.1	0.688	79.7 ± 33.2	81.6 ± 35.6	0.837	70.6 ± 33.6	65.3 ± 32.6	0.098	78.2 ± 26.6	82.9 ± 22.0	0.329
≥130	7.4	6.3	9.0	9.0		16.7	16.7		4.5	1.5		5.6	16.7		9.0	4.5		0.0	0.0	
HDL cholesterol (mg/dL)	52.6 ± 16.1	47.4 ± 14.3	50.8 ± 12.3	47.6 ± 14.1	0.041	55.2 ± 24.0	38.6 ± 11.3	0.005	52.6 ± 14.5	49.3 ± 13.7	0.012	58.1 ± 27.4	45.2 ± 16.5	0.060	51.4 ± 14.3	47.5 ± 14.3	0.000	55.1 ± 17.2	50.4 ± 16.2	0.113
<40	18.4	32.0	17.9	32.8		16.7	44.4		16.4	22.4		27.8	50.0		19.4	31.3		15.8	36.8	
Blood glucose (mg/dL)	149.3 ± 58.2	141.1 ± 47.2	155.4 ± 80.9	143.3 ± 46.3	0.232	140.4 ± 29.5	160.5 ± 82.3	0.303	147.1 ± 44.4	140.2 ± 41.0	0.137	157.3 ± 38.4	149.6 ± 39.2	0.161	146.9 ± 53.0	135.9 ± 46.3	0.020	144.6 ± 62.2	128.3 ± 33.2	0.040
Systolic blood Pressure	140.9 ± 21.9	140.5 ± 19.8	143.2 ± 21.2	143.8 ± 20.3	0.807	135.6 ± 26.7	136.1 ± 19.9	0.923	142.7 ± 21.9	139.4 ± 19.6	0.106	139.4 ± 19.6	142.3 ± 15.8	0.354	139.4 ± 0.2	141.4 ± 21.0	0.391	138.6 ± 28.6	133.1 ± 11.9	0.311
≥140	50.8	49.6	61.2	55.2		50.0	55.6		43.3	7.5		50.0	22.2		49.3	10.4		47.4	0.0	
Diastolic blood Pressure	75.8 ± 13.5	76.9 ± 13.2	77.6 ± 15.4	79.4 ± 12.3	0.361	73.6 ± 14.6	72.9 ± 14.6	0.833	75.1 ± 12.8	77.8 ± 16.9	0.644	76.0 ± 13.1	74.5 ± 9.4	0.266	75.6 ± 11.7	77.5 ± 13.4	0.266	72.8 ± 0.1	75.2 ± 17.3	0.491
≥90	11.7	13.7	16.4	14.9		16.7	11.1		46.3	9.0		66.7	5.6		47.8	20.9		26.3	10.5	
Hemoglobin (g/dL)	12.2 ± 2.0	12.1 ± 1.9	11.8 ± 2.2	12.0 ± 2.0	0.457	13.0 ± 2.2	11.9 ± 2.1	005	12.1 ± 2.0	12.0 ± 1.7	0.683	12.1 ± 1.7	11.3 ± 2.2	0.100	12.7 ± 2.1	12.8 ± 1.7	0.758	12.1 ± 1.3	11.7 ± 1.6	0.623
Anemia	47.3	39.5	58.2	44.4		22.2	44.4		50.7	37.3		61.1	12(66.7)		38.8	29.9		36.8	42.1	
C-reactive protein (mg/dL)	0.5 ± 0.1	0.7 ± 0.2	0.51 ± 1.0	0.66 ± 0.1	0.688	0.50 ± 0.0	0.7 ± 0.2	0.903	0.50 ± 0.0	0.73 ± 0.3	0.717	0.51 ± 0.0	0.70 ± 0.1	0.129	0.53 ± 0.2	0.69 ± 0.3	0.441	0.50 ± 0.0	0.65 ± 0.2	0.820

DII score range: Tertile 1 (−0.817~1.308), Tertile 2 (1.310~2.572), Tertile 3 (2.578~6.553). The parentheses are the pre–post mean difference results. Data are reported as mean ± standard deviation for continuous variables and as percentages for categorical variables. *p*-value was calculated by Student’s *t*-test or Mann–Whitney U test for continuous variables and by chi-squared test or Fisher’s exact test for categorical variables as appropriate.

### 3.5. Factors Influencing DII Score

In Tertile 1, the improvement in DII score was related to the intake of carbohydrates, protein, and fat, and in Tertile 3, it was related to the intake of energy, carbohydrates, protein, and fat, and nutritional status (Table 5). 

## 4. Discussion

Over one year, we examined the balance and adequacy of nutrient intake among older adults and investigated the effectiveness of the DII and senior-friendly quality foods to improve nutritional status. South Korea has an exceptionally rapidly aging population compared to other countries. Our 256 participants had an average age of 82.5 years, and 55.1% were 75–84 years of age. Thus, South Korea has one of the highest rates of population aging and is the fastest aging of the OECD countries.

The BMI of the participants was 23.3 kg/m^2^, similar to that reported by Seo et al. [24], who examined the impact of BMI on the physical fitness of the elderly in South Korea, and by He et al. [25], who investigated the relationship between serum CRP levels and senile hypertension. Impairments to ADL, which reflect the physical health status of the elderly, tend to increase with age, indicating a decline in physical function with aging [26]. The ADL assessment in this study was conducted to alleviate difficulties in daily life among the elderly and to identify disparities in perceptions of programs and services [27]. The participants scored 8.3 points, which is similar to the findings by Lee et al. [28] on determinants of ADL performance among the elderly in urban and rural areas. This indicates that the participants could independently manage their daily lives, as evidenced by the high rate of participants living alone (73.8%). Gobbens et al. [29] reported an ADL score of 15.3, somewhat higher than in this study. This difference may be because they evaluated participants ≥75 years of age with >50% marriage or cohabitation rates.

According to the 2020 “Survey on the living conditions of the elderly”, which provides data for social policies aimed at improving the quality of life of the elderly, the average elderly person is 73.8 years of age with an average of 1.9 chronic diseases [30]. In this study, participants had an average of 2.5 chronic conditions. This may result from their advanced age, and the fact that many received community-based elderly care, indicating a higher prevalence of disease than in the general population. Aging leads to deteriorating dental health, reduced gastrointestinal function, and age-related declines in taste and smell, resulting in reduced appetite in old age. Therefore, maintaining an enjoyable and nutritious diet is crucial for maintaining good nutritional status in the elderly. In December 2017, the Ministry of Agriculture, Food, and Rural Affairs of South Korea established standards for senior-friendly industries. This has led to the development of customized products for the elderly that adjust factors such as meal texture and viscosity to improve intake, digestion, and absorption [31].

Currently, 175 products from 27 companies have been selected as excellent foods for the elderly [32]. In this study, the intervention group received ≥1 modified side dish with each meal, and the control group received regular meals. There was no significant difference in nutrient intake between groups and both showed an increase in their nutrient intake. Senior-friendly foods are products that combine raw materials or have added nutrients to ensure the intake of ≥3 essential nutrients—including protein, vitamin A, vitamin C, vitamin D, riboflavin, niacin, calcium, potassium, and dietary fiber—up to ≥10% of the recommended or adequate level for adult males aged 65–74 years, according to South Korean nutrient intake standards [33].

There was a significant increase in energy, protein, and fat intake in the intervention group. Age-friendly foods that contain balanced amounts of nutrients are reported to provide more nutrients per unit of food consumption [34]. Before the intervention, the rates of the recommended or adequate intake were low, but after the intervention, the intakes of many nutrients that met the requirements increased. However, despite the significant increase in vitamin D intake in the intervention and control groups, it was lower than the recommended intake. Vitamin D is not only involved in bone and mineral homeostasis, but also has extra-skeletal functions [35]. According to the Korean Centers for Disease Control and Prevention [36], vitamin D intake varies with age, with peaks in people aged 30–49 and 50–64 years. However, vitamin D intake was insufficient in all age groups, at <50% of the recommended level. In particular, participants ≥65 years of age, who had a low intake of animal-based foods, had a vitamin D intake <20% of the recommended level. This indicates little difference between the vitamin D intake of the study participants and the reported insufficiency in the population. The United Kingdom National Diet and Nutrition Survey reported low vitamin D intake among community-dwelling older adults [37]. Therefore, efforts should be made to develop age-appropriate foods that meet the nutrient requirements of the elderly.

The participants showed a slight general improvement in the DETERMINE score. Significant improvements in the DETERMINE score were observed in the intervention group. Among the low-, moderate-, and high-risk groups, the improvement in the DETERMINE score was greatest in Tertile 3, decreasing from 68.7% to 46.3% (indicating slight improvement). Therefore, the DETERMINE dietary assessment tool is suitable for use in groups with low DII scores. According to Sadarangani et al. [38], the mean rate of moderate-to-high risk of nutritional inadequacy in the Asian Pacific Islander population is 40.65%, similar to the 38.01% observed after the intervention in Tertile 3. This suggests that the DETERMINE tool can be used for dietary risk assessment in Asians. All 256 participants showed overall improvements in nutritional management after participating in a two-month nutrition intervention program for the elderly.

Improvements were observed in the NQ-E in relation to the nutrition, balance, variety, moderation, and dietary behavior scores of the participants. However, compared with the nutrition index for older adults described by Choi et al. [39], all scores were lower except for the moderation score. This could result from the higher proportion of older adults living alone and the higher mean age in this study, which resulted in lower mean scores for diet quality and nutritional status. In addition, the moderation score was higher because participants with limited mobility and low income had limited access to a variety of foods. Although not mentioned in this study, it is important to consider that older adults in the community have a higher prevalence of food insecurity, a limited consumption of snacks outside of main meals, and an inadequate consumption of fruit and dairy products. Taking these factors into account, it may be beneficial to provide tailored meals that emphasize variety and balance after assessing the dietary habits and intake statuses of individuals.

The use of senior-friendly quality foods resulted in a significant improvement in DII scores after the intervention. The group with a better pre-DII score (Tertile 1) showed greater improvement than those with worse scores (Tertiles 2–3). Furthermore, the intervention and control groups in Tertile 3 showed slight improvements (scores of −5.1 and −3.8, respectively). This suggests that individuals with poor pre-DII scores could further improve their DII scores and were more interested in nutrition education. In a Japanese study, Kotemori et al. [40] reported that the DII score for women was −1.01. Japanese participants, 40–69 years of age and undergoing cancer screening, achieved similar scores after the intervention. In a study in China, the average DII score for postmenopausal women was +0.89; therefore, the preintervention DII score of the participants was higher than that of postmenopausal women [41]. Because higher DII values are associated with lower lumbar and total hip bone densities and increased risks of osteoporosis and fractures, this study in older adults showed higher DII values than postmenopausal women [42].

Triglycerides significantly improved in the intervention group, particularly in Tertile 3. Similarly, although all groups showed improvements in blood glucose levels, significant improvement was only observed in Tertile 3. This is similar to the postintervention value in the control group reported by Shin et al. [43]. CRP is produced mainly by the liver in response to inflammatory stimuli [44]. The mean CRP level increased from 0.5 to 0.7 mg/dL, within the normal range. We estimated that the subjects’ level of inflammation was 90.2% of the normal range before the intervention and did not change significantly after the intervention.

In the high- (Tertile 3), compared to the low-DII-score group (Tertile 1), there were lower intakes of energy, carbohydrates, protein, and fat. Protein is important for muscle strength, immune function, and energy production. Jang et al. [45] reported positive associations between energy and protein intake and muscle strength. This suggests that adequate nutrient intake could reduce the DII score, boost the immune system, and promote metabolic health.

This study has methodological limitations as it is a cross-sectional study, making it impossible to assess the elderly individuals over time. However, we systematically investigated the dietary inflammatory index (DII) of individuals aged 65 and above. Research targeting individuals over 65 years old is uncommon in South Korea, particularly studies comparing data from before and after clinical examinations. Additionally, the clinical marker indicators obtainable from the study participants were limited; markers such as TNF, IL-6, and uric acid were not included. Despite the small sample size of our study and the absence of data on physical activity and waist circumference, our findings provide insights into the suitability of senior-friendly diets for South Korea’s rapidly aging society. Moreover, focusing on a specific region’s elderly population prevents generalization to the entire elderly population of Korea. Nonetheless, we emphasize that this study can contribute to raising awareness among older adults about the importance of health.

## 5. Conclusions

The present results indicate that an elderly-friendly diet can have a significant impact on nutritional status and health improvement in older people who typically have a low-quality diet and a poor DII.

## Figures and Tables

**Table 1:** Characteristics of study participants at baseline.

Variables	Total N = 256	Intervention Group N = 201	Control Group N = 55	*p*-Value
Age (years)	82.49 ± 6.0	82.5 ± 6.0	82.6 ± 6.1	0.937
65–74	17 (6.6)	13 (6.5)	4 (7.3)	0.977
75–84	141 (55.1)	111 (55.2)	30 (54.5)	
≥85	98 (38.3)	77 (38.3)	21 (38.2)	
Gender				
Men	86 (33.6)	68 (33.8)	18 (32.7)	0.878
Women	170 (66.4)	133 (66.2)	37 (67.3)	
Family status				
Living alone	189 (73.8)	154 (76.6)	35 (63.6)	0.052
Family together	67 (26.2)	47 (23.4)	20 (36.4)	
Education				
No education	113 (44.1)	91 (45.3)	22 (40.0)	0.442
Elementary school	74 (28.9)	57 (28.4)	17 (30.9)	
Middle school	27 (10.5)	23 (11.4)	4 (7.3)	
High school	30 (11.7)	20 (10.0)	10 (18.2)	
≥University	12 (4.7)	10 (5.0)	2 (3.6)	
Monthly income (KRW)				
3–5 million	2 (0.8)	0 (0.0)	2 (3.6)	0.060
2–3 million	5 (2.0)	3 (1.5)	2 (3.6)	
1–2 million	14 (5.5)	10 (5.0)	4 (7.3)	
<1 million	216 (84.4)	173 (86.1)	43 (78.2)	
No income	19 (7.4)	15 (7.5)	4 (7.3)	
Alcohol consumption				
Drinking	32 (12.5)	21 (10.4)	11 (20.0)	0.058
Non-drinking	224 (87.5)	180 (89.6)	44 (80.0)	
Smoking				
Smoking	14 (5.5)	12 (6.0)	2 (3.6)	0.500
Non-smoking	242 (94.5)	189 (94.0)	53 (96.4)	
Body mass index (kg/m^2^)	23.3 ± 3.7	23.4 ± 3.7	22.8 ± 3.7	0.274
<18.5 (underweight)	25 (9.8)	19 (9.5)	6 (10.9)	0.755
18.5–22.9 (normal)	98 (38.3)	76 (37.8)	22 (40.0)	
23.0–24.9 (overweight)	53 (0.7)	40 (19.9)	13 (23.6)	
≥25 (obesity)	80 (31.3)	66 (32.8)	14 (25.5)	
K-ADL	8.3 ± 2.1	8.3 ± 2.3	8.1 ± 1.4	0.325
Number of chronic diseases	2.5 ± 1.5	2.6 ± 1.4	2.5 ± 1.6	0.767

Korean Instrumental Activities of Daily Living (K-IADL) Scale. Data are reported as mean ± standard deviation for continuous variables and as percentages for categorical variables. *p*-value was calculated by Student’s *t*-test or Mann–Whitney U test for continuous variables and by chi-squared test or Fisher’s exact test for categorical variables as appropriate.

**Table 5:** Multiple regression analyses of factors associated with DII score.

Variables		Tertile 1			Tertile 2			Tertile 3		
		B	β	*t*	B	β	*t*	B	β	*t*
Nutrients	Energy	−0.003	−0.512	−1.886	0.002	0.323	1.085	−0.012	0.573	−4.527 ***
	Carbohydrate	0.022	0.552	2.735 **	−0.008	−0.206	−0.983	0.054	0.795	4.613 ***
	Protein	−0.036	−0.269	−2.552 *	−0.037	−0.233	−1.992	−0.026	−0.231	−2.642 **
	Fat	0.094	0.672	3.763 ***	0.025	0.180	0.912	0.147	0.142	4.470 ***
Nutritious Status	DETERMINE	−0.009	−0.030	−0.217	−0.110	−0.321	−2.643 **	−0.086	−0.315	−3.211 **
	F	3.772			3.997			6.721		
	R^2^	0.595			0.440			0.717		

Multiple regression analysis was performed to identify the factors that influence DII score of elderly adults, and *p*-values of <0.05 were used to denote statistical significance. Indication; * *p* < 0.05, ** *p* < 0.01, *** *p* < 0.001.

## Data Availability

The data that support the findings of this study are available from the funding institute upon reasonable request.
